# Genetic diversity of *Salix koreensis* based on inter-simple sequence repeat (ISSR) in South Korea

**DOI:** 10.1186/1753-6561-5-S7-P15

**Published:** 2011-09-13

**Authors:** Jae In Park, Go Eun Choi, Jae Ik Nam, Young Mi Kim

**Affiliations:** 1 Chungbuk National University, Korea; 2 Chungbuk National University, Korea Forest Research Institute, Korea

## Background

*Salix koreensisis* native to Korea, Japan and China, is a deciduous broadleaved tall tree growing throughout Korea. This species is dioecius and planted along the streets and for landscaping. Along the rivers the species grows well reaching up to 20 meters in height. Trees bloom in April and fruits ripe in May. This study was carried out to investigate the genetic diversity of *S. koreensis* within and among 10 populations throughout South Korea using ISSR marker analysis.

## Materials and methods

From 10 populations identified throughout the country 8 to 11 individual trees per population were sampled, totalling 84 individual trees.Twigs with leaves were collected and used as material for genetic analysis. DNA extraction was performed using GENE ALL plant SV DNA purification Kit (Gean all, Korea) and amplified by PCR using 5 UBC (University of British Columbia) primers (#807, #834, #842, #856, #881) which showed good results in previous experiments. PCR products were electrophoresed and gels photographed. Amplified fragments were visually scored as present (1) or absent (0). Observed and effective numbers of alleles, Shannon's information index, number of polymorphic loci, Nei's genetic distance were estimated using POPGENE 3. and genetic distance was analysed using UPGMA.

## Results and conclusions

The overall number of polymorphic ISSR amplicons was 80 and mean per primer was 16. The percentage of polymorphic loci was as follows: Namwon population had the highest level (61.25%), Seomjin river population had the lowest level (37.50%) and the overall mean was 48 %. Shannon’s Information Index indicating genetic diversity ranged from 0.17 (Seomjin river population) to 0.28 (Hanam population) with a mean of 0.24. Shannon’s information index of this species was lower than the one found in other species. This result is likely due to the fact that *S. koreensis* is frequently propagated by asexual reproduction. The UPGMA dendrogram based on Nei’s genetic distance did not show any clear geographic pattern. The Seomjin river population with low genetic diversity was genetically more distant from the other populations and the Upo population was the next more distant one. Hanam and Yangchon populations were the ones more genetically related.

